# Identification of spatio-temporal clusters of lung cancer cases in Pennsylvania, USA: 2010–2017

**DOI:** 10.1186/s12885-022-09652-8

**Published:** 2022-05-17

**Authors:** Nuria Camiña, Tara L. McWilliams, Thomas P. McKeon, Trevor M. Penning, Wei-Ting Hwang

**Affiliations:** 1 Center of Excellence in Environmental Toxicology, Perelman School of Medicine, University of Pennsylvania, Philadelphia, PA USA; 2 Department of Systems Pharmacology & Translational Therapeutics, Perelman School of Medicine, University of Pennsylvania, Philadelphia, PA USA; 3 Center for Clinical Epidemiology and Biostatistics, Perelman School of Medicine, University of Pennsylvania, Philadelphia, PA USA; 4 Department of Geography, Temple University, Philadelphia, PA USA; 5 Abramson Cancer Center, Perelman School of Medicine, University of Pennsylvania, Philadelphia, PA USA; 6 Department of Biostatistics, Epidemiology and Informatics, Perelman School of Medicine, University of Pennsylvania, Philadelphia, PA USA

**Keywords:** Lung cancer, Incidence, Spatio-temporal, Geographic clustering, Scan statistics, Pennsylvania

## Abstract

**Background:**

It is known that geographic location plays a role in developing lung cancer. The objectives of this study were to examine spatio-temporal patterns of lung cancer incidence in Pennsylvania, to identify geographic clusters of high incidence, and to compare demographic characteristics and general physical and mental health characteristics in those areas.

**Method:**

We geocoded the residential addresses at the time of diagnosis for lung cancer cases in the Pennsylvania Cancer Registry diagnosed between 2010 and 2017. Relative risks over the expected case counts at the census tract level were estimated using a log-linear Poisson model that allowed for spatial and temporal effects. Spatio-temporal clusters with high incidence were identified using scan statistics. Demographics obtained from the 2011–2015 American Community Survey and health variables obtained from 2020 CDC PLACES database were compared between census tracts that were part of clusters versus those that were not.

**Results:**

Overall, the age-adjusted incidence rates and the relative risk of lung cancer decreased from 2010 to 2017 with no statistically significant space and time interaction. The analyses detected 5 statistically significant clusters over the 8-year study period. Cluster 1, the most likely cluster, was in southeastern PA including Delaware, Montgomery, and Philadelphia Counties from 2010 to 2013 (log likelihood ratio = 136.6); Cluster 2, the cluster with the largest area was in southwestern PA in the same period including Allegheny, Fayette, Greene, Washington, and Westmoreland Counties (log likelihood ratio = 78.6). Cluster 3 was in Mifflin County from 2014 to 2016 (log likelihood ratio = 25.3), Cluster 4 was in Luzerne County from 2013 to 2016 (log likelihood ratio = 18.1), and Cluster 5 was in Dauphin, Cumberland, and York Counties limited to 2010 to 2012 (log likelihood ratio = 17.9). Census tracts that were part of the high incidence clusters tended to be densely populated, had higher percentages of African American and residents that live below poverty line, and had poorer mental health and physical health when compared to the non-clusters (all *p* < 0.001).

**Conclusions:**

These high incidence areas for lung cancer warrant further monitoring for other individual and environmental risk factors and screening efforts so lung cancer cases can be identified early and more efficiently.

## Introduction

Lung cancer is the most frequently diagnosed cancer worldwide, accounting for 1.74 million deaths annually and lung cancer cases are expected to increase by 38% to 2.89 million by 2030 [1]. Lung cancer is also the leading cause of cancer mortality in both men and women in the U.S. In 2021, Pennsylvania (PA) was ranked 32 out of 49 states with an age-adjusted lung cancer incidence rate of 63 per 100,000 population and a five-year survival rate of 25 percent [2]. An estimated 5,990 Pennsylvanians are expected to die from lung cancer in 2022 with approximately 11,170 new cases being reported [3]. People diagnosed at early stages of lung cancer are five times more likely to survive; however, in Pennsylvania only 16 percent of lung cancer cases are diagnosed at early stages [2].

Documenting the extent of cancer incidence remains central to improving public health research and to developing population-based strategies for cancer prevention. The need to understand the incidence of lung cancer is influenced by potentially modifiable risk factors (e.g., tobacco use, alcohol drinking, unhealthy diet, radon exposure) and others that are not (e.g., inherited genetic mutations) [3]. Cancer outcomes are influenced also by socioeconomic status, access to care, supportive services, and rural–urban environmental factors, all of which contribute to both the physical and mental health of cancer patients [4].

Mapping spatial patterns of lung cancer risk is an increasingly popular approach given the greater availability of geographically enabled cancer data and sophisticated visualization methods [5]. Maps are useful for examining disease patterns in relation to local environmental factors with the ability to examine disease causation through the identification of demographic patterns and trends [6, 7].

Spatial statistical methods like space–time models can also be used to quantify patterns and trends over space and time (i.e., spatio-temporal) and cancer clusters are frequently used by researchers to respond to public concerns. The aims of this study were to examine spatio-temporal patterns of lung cancer incidence in Pennsylvania over an 8-year period (2010–2017), identify high incidence clusters, and compare the demographic and health characteristics of residents inside and outside of clusters.

## Methods

### Data sources

Lung and bronchus cancer cases in PA between 2010 and 2017 were obtained from the Pennsylvania Cancer Registry (PCR) [8] using International Statistical Classification of Diseases, 10th revision (ICD 10) diagnosis codes—C340 (main bronchus), C341 (upper lobe, bronchus or lung), C342 (middle lobe, bronchus or lung), C343 (lower lobe, bronchus or lung), C348 (overlapping sites of bronchus and lung), and C349 (unspecified part of bronchus or lung). PCR is an incidence-based registry and has earned Gold Certification from the North American Association of Central Cancer Registries (NAACCR), the highest level of data quality achieving at least 95% completeness, for all years under study [9]. The following three exclusion criteria were applied to exclude cases that were: (i) in situ and non-carcinoma histology, (ii) not uniquely matched with a census tract ID, and (iii) the age of diagnosis belonged to an age group with zero population size as estimated by US Census Bureau indicating a possible error. This resulted in a total of 73,937 cases from 3,197 census tracts. We used the census tract, the small and relatively permanent statistical subdivision defined by the US Census Bureau, as the unit of analysis for the consistency in the data collected over the years and the validity when used in research studies [10]. We conducted the present analysis under a data use agreement with the Pennsylvania Department of Health and with the approval of the University of Pennsylvania Institutional Review Board (IRB number 831671).

The reported street addresses at the time of diagnosis were geocoded using ArcGIS 10.6.1 software [11] and matched with the 2010 census tract ID. Lung cancer cases were grouped into 18 age groups (0–4, 5–9, 10–14,15–19, 20–24, 25–29, 30–34, 35–39, 40–44, 45–49, 50–54, 55–59, 60–64, 65–74, 75–84, 85 and above). Annual population size for the same year by age groups for a census tract was obtained using the American Community Survey (ACS), a national survey conducted by the US Census Bureau that provides various individual demographic and household information on a yearly basis [12].

Demographic data at the census tract level were extracted from the 2011–2015 ACS including median age (years), percentage of males, distribution by race and ethnicity, per capita income, median household income (thousands of $), percent poverty, distribution by educational attainment, total population size, and population density (per square mile).

Poor mental health and poor physical health, defined as the percent of individuals ≥ 18 years who self-reported having 14 or more days during the past 30 days in which their mental or physical health was not good, were extracted from the Centers for Disease Control and Prevention (CDC) PLACES 2020 database derived using the 2018 Behavioural Risk Factor Surveillance System (BRFSS). Both mental and physical health measures were based on self-assessment only without an objective health component [13].

### Age-adjusted Incidence rates and trends over time

The age-adjusted incidence rates (number of cases per 100,000) for each census tract were calculated by adjusting the crude incidence rate with respect to the 2000 U.S. Standard Million Population, a commonly used standard population for adjustment that assumes a total population of 1,000,000 [14]. The adjustment used the 18 age groups and population size estimates described above. A choropleth map for the age-adjusted incidence rate using the cumulative cases over 8 years was created to visualize the spatial pattern. Temporal trends in the adjusted incidence rates were examined and modeled using linear quantile mixed models [15]. Such mixed models were utilized to allow census tract level random effects of intercept and slope for the calendar year to be estimated, while the use of the quantile regression provided a robust summary of the trends that were less sensitive to outlying values in the incidence rates, which are often observed in smaller census tracts. The estimated 50^th^ (median), 75^th^, 80^th^, and 90^th^ quantiles were plotted, and the mean profile was included as a reference.

### Spatio-temporal disease risk and mapping

To understand the spatio-temporal disease risk, we modeled the observed case counts through a log-linear Poisson regression with both spatial and temporal terms, as well as a space–time interaction term. Specifically, the mean case count for location *i* (in this case a census tract) and year *j* was modeled as the expected case counts for the same location and year combination (E_*ij*_) times the relative risk parameter, RR_*ij*_, which is also indexed by location *i* and year *j* (i.e., relative risk specific to a location and a time). The expected case counts E_*ij*_ were determined based on the age distribution of the corresponding location *i* and year *j* such that E_*ij*_ equals the crude incidence rate in a particular age group in the study population in year *j* times the population size in the same age group of the location *i* from the same year (i.e., internal standardization). Extending the model proposed by Lawson et al. [16] for the spatial model, the log of the space–time relative risk parameter RR_*ij*_ was modeled with four components: an intercept as the overall relative risk for the study region, location-specific random effects, a linear trend term in time *j*, and the interaction random effects between the location and time. The spatial random effects were assumed to follow a normal distribution under the conditional autoregressive (CAR) setting based on Queen contiguity spatial weight matrix (i.e., two areas are considered neighbors if they share a common boundary). The model was fit using the R-integrated nested Laplace approximation, R-INLA [17] under the Bayesian framework with a normal prior distribution. Temporal trends in the RR estimates from 20 randomly selected census tracts were plotted to examine the changes over time. The spatial pattern for the estimated RR for a given year was illustrated using a choropleth map. Furthermore, we calculated the standardized incidence ratio (SIR) for each census tract as the total number of cases observed divided by the total number of cases expected E_*ij*_ across the 8 years combined. We then created a choropleth map of the SIR to examine this spatial pattern empirically with SIR > 1 indicating an elevated risk such that the number of cases observed is higher than the expected number of cases.

### Detection of high-risk clusters

We used the SaTScan cluster detection method which employs Kulldorff scan statistics to detect high risk clusters. This approach has been widely used in spatial statistics to evaluate the risk of disease geographically to detect high risk clusters. This method generated circular spatial windows of various sizes and evaluated the observed over the expected number of cases by comparing inside versus outside the circles to identify statistically significant clusters [18]. To detect spatio-temporal clusters [19, 20], scan statistics covered the study area with many overlapping “windows” now defined as cylinders with the base as the area and the height as the time period in the space–time setting. As the window expanded to contain more areas and more cases, we used a log-linear ratio (LLR) to compare the number of cases inside the windows to the number of cases outside the window. The null hypothesis was calculated under the probability that being a case is the same inside and outside the window relative to the age-adjusted expected number of cases. A LLR >  > 1 indicated evidence that the current window forms a high incidence or high-risk cluster. In our analysis, the age-adjusted expected case counts used were the same E_*ij*_ that was used for the log-linear Poisson model in the previous section. The most likely cluster (i.e., the window with the maximum LLR) and secondary clusters (i.e., other statistically significant windows at 0.05 significance level) were identified in the current analysis. The RR of each cluster was determined by the total number of cases observed over the total number of cases expected in the years when the cluster is present. The statistical significance of a cluster was determined through a Monte Carlo hypothesis testing procedure [21]. The proposed analysis was performed using the R shiny application SpatialEpiApp, which allows estimation of spatio-temporal disease risk and detection of clusters [22].

### Comparison of census tracts in high-risk cluster versus not

The nonparametric Wilcoxon rank sum test with a continuity correction was used to compare demographic variables between census tracts in any high incidence cluster at any time during 2010 to 2017 versus those not in any clusters. Data on smoking, which is a known risk factor for lung cancer development, were not available at the census tract level and for the same time frame as the demographic variables used, thus comparison of the smoking prevalence between census tracts was not possible. A two-sided *p* < 0.01 was considered statistically significant. We used a lower *p*-value threshold for statistical significance to account for testing multiple variables.

## Results

### Age-adjusted incidence rates and spatio-temporal disease risk

**Fig. 1:**
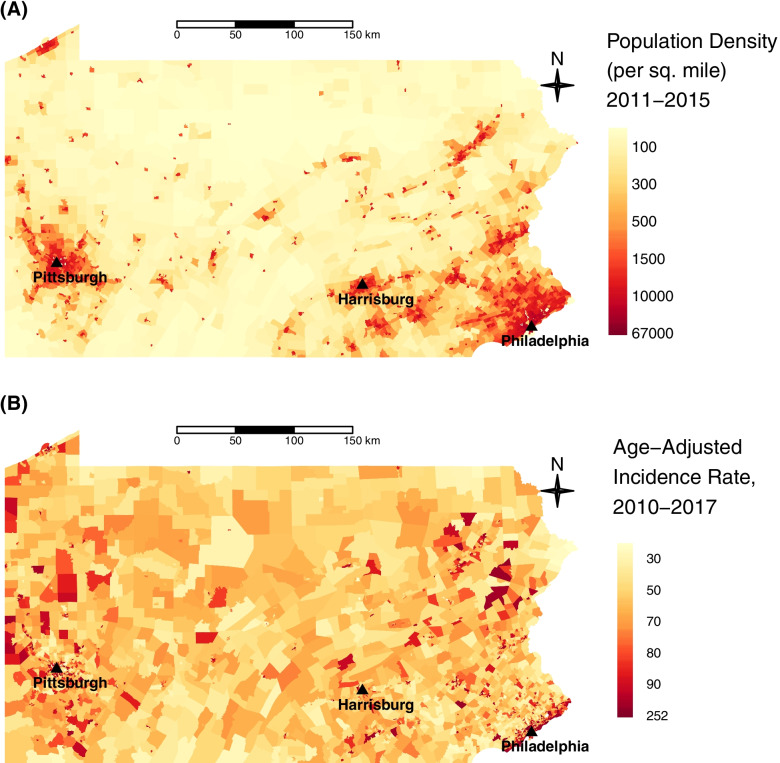
**A** population density based on the 2011–2015 5-year ACS, **B** age-adjusted incidence rate based on the cumulative cases over 8 years from 2010 to 2017

**Fig. 2:**
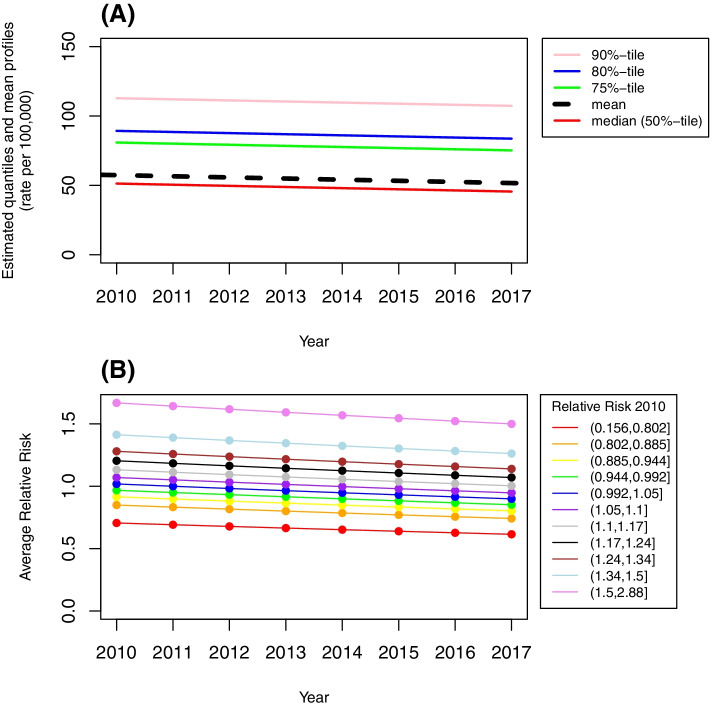
**A** temporal trends in the estimated quantiles and mean profiles (rate per 100,000) from 2010 to 2017 based on the linear mixed quantile regression model, **B** temporal trends in the average of the estimated RR from 2010 to 2017 based on the log-linear Poisson spatio-temporal model, grouped by decile of 2010 estimates

The population density by census tract in Pennsylvania using the 2011–2015 ACS is shown in Fig. 1A. The population was mainly concentrated in a small number of metropolitan areas including the southeast and western regions of Pennsylvania, specifically in Philadelphia and Pittsburgh areas, respectively. A map of age-adjusted incidence rate using the cumulative cases over 8 years is provided in Fig. 1B showing that higher age-adjusted incidence rates were mainly observed in the major cities located in southeastern (e.g., Philadelphia), northeastern (e.g., Allentown, Scranton), and western (e.g., Pittsburgh, Erie) Pennsylvania. The age-adjusted incidence rates decreased slightly over the study period with median incidence rates (25^th^-75^th^ quantiles) of 51.7 per 100,000 (25.2 to 83.3) for 2010, 49.1 per 100,000 (24.4 to 78.9) for 2013, and 45.3 per 100,000 (22.0 to 72.3) for 2017, respectively, approximately 0.8 per 100,000 per year, for all the quantiles and the mean values are shown in Fig. 2A.

**Fig. 3:**
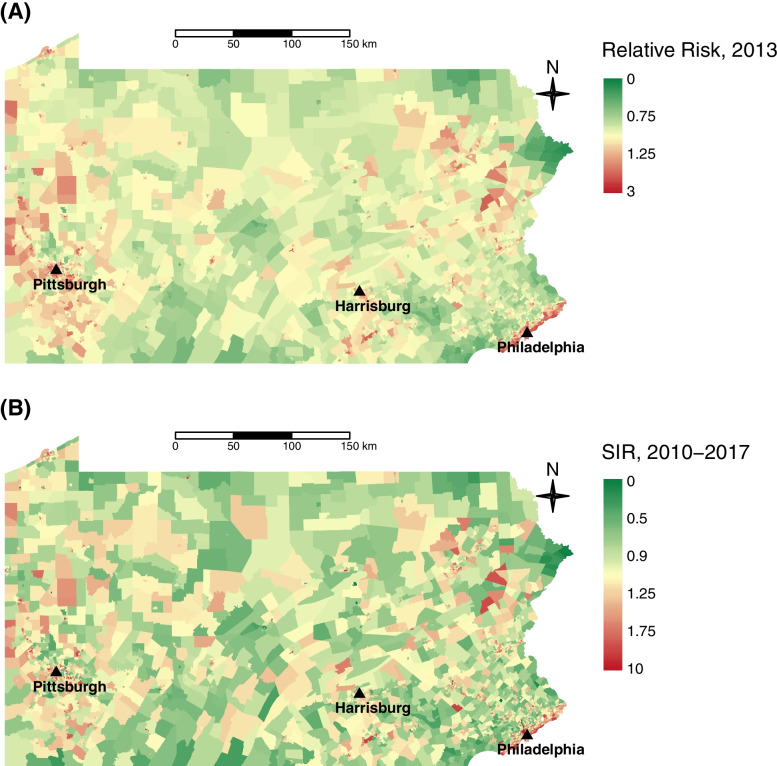
**A** estimated RR for 2013 based on log-linear Poisson spatio-temporal model, **B** SIR based on the cumulative cases from over 8 years from 2010 to 2017

The estimated relative risk (RR) from the log-linear Poisson regression model suggested no statistically significant space and time interaction (*p* > 0.05) and revealed a steady decrease in lung cancer incidence from 2010 to 2017. The median RR values (25^th^-75^th^ quantiles) were 1.07 (0.93 to 1.26) for 2010, 1.01 (0.88 to 1.19) for 2013, and 0.95 (0.82 to 1.12) for 2017, respectively. Figure 2B shows the estimated RR over time for 20 randomly selected census tracts and the median of RR estimates for a decile group created using the 2010 estimates. The parallel lines observed in Fig. 2A and B reflected that the fitted models suggested no space and time interaction such that the decreasing trends in the age-adjusted incidence rates and RR values were consistent across the study region. Maps showing the estimated RR for 2013 and SIR are provided in Fig. 3A and B, respectively, indicating a similar pattern to the age-adjusted incidence rates as shown in Fig. 1B, such that higher values of RR and SIR were concentrated in the major cities located in southeastern (e.g., Philadelphia), northeastern (e.g., Allentown, Scranton) and western (e.g., Pittsburgh, Erie) Pennsylvania while most of the central PA showed lower than expected case counts (RR < 1 and SIR < 1). Maps from other years also show a similar pattern (maps not shown).

### Detection of high-risk clusters

**Fig. 4:**
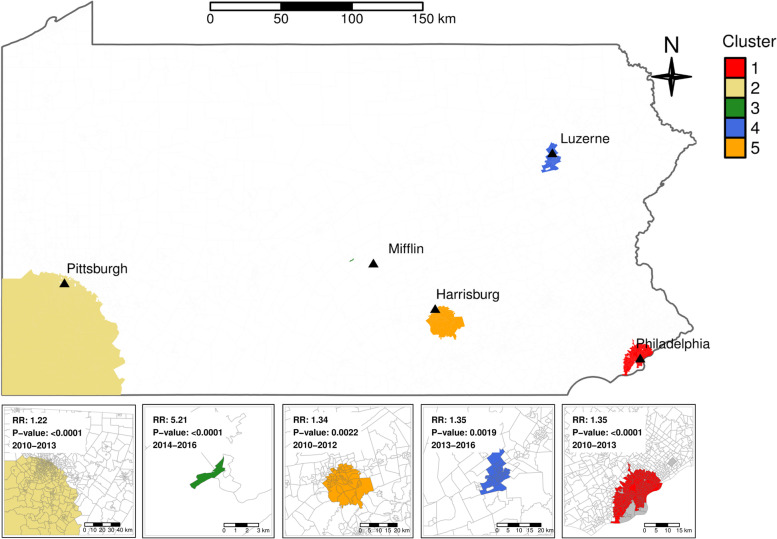
Five spatio-temporal clusters in PA and the associated RRs and *p*-values. Cluster 3 shows Mifflin County; Cluster 1 Delaware, Montgomery, and Philadelphia Counties; Cluster 2 Allegheny, Fayette, Greene, Washington, and Westmoreland Counties; Cluster 4 Luzerne County; and Cluster 5 Dauphin, Cumberland, and York Counties

**Fig. 5:**
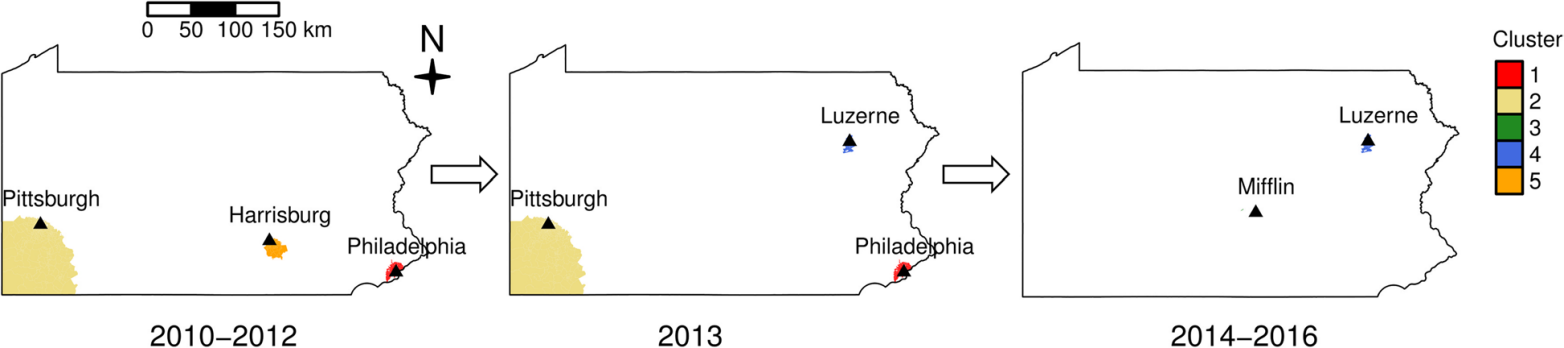
The locations of the spatio-temporal clusters identified in PA for 2010 – 2012, 2013, and 2014 – 2016, respectively

Five spatio-temporal clusters were identified based on lung cancer cases in Pennsylvania during the study period 2010 to 2017, as shown in Fig. 4. Information for each of the clusters is provided in Table 1. The most likely cluster (Cluster 1), which is the cluster with the largest LLR, was from 2010 to 2013 with a RR of 1.35. This cluster with an average population size of 1,276,868 was in the Philadelphia metropolitan area including the neighboring Delaware and Montgomery Counties, part of the southeastern PA. Among the four secondary clusters, one cluster (Cluster 2) from 2010 to 2013 with a RR of 1.22 was in southwestern PA: Allegheny County, Fayette County, Greene County, Washington County, and Westmoreland County. This cluster had the highest number of observed lung cancer cases reaching 4,601. Three other secondary clusters (Clusters 3 to 5) were identified for varying periods: Cluster 3 was in Mifflin County in the central PA from 2014 to 2016, associated with the smallest number of individuals 3,772 on average, and observed a total of 30 cases while only 6 cases were expected; Cluster 4 was in Luzerne County from 2013 to 2016 near the Allentown-Scranton region; lastly, Cluster 5 was in the southcentral PA region near the Harrisburg area from 2010 to 2015 that included Dauphin, Cumberland, and York Counties. It is important to note that the size of the area covered by each cluster differed significantly, and the location and the numbers of identified clusters also varied from one time period to another. For example, as shown in Fig. 5, there were three clusters (Clusters 1, 2, 5) from 2010 to 2012; three clusters (Clusters 1, 2, 4) in 2013, and two clusters (Clusters 3 and 4) from 2014 to 2016. No clusters were identified in 2017, the final year of the study period.

**Table 1:** Results of cluster analysis of lung cancer cases in Pennsylvania developed between 2010 and 2017

Cluster	Averaged population size	Years Detected	County	Observed cases	Expected cases	RR	LLR
1	1,276,868	2010–2013	Delaware, Montgomery and Philadelphia	3,557	2,676	1.4	136.6
2	1,260,363	2010–2013	Allegheny, Fayette, Greene, Washington and Westmoreland	4,601	3,823	1.2	78.6
3	3,772	2014–2016	Mifflin	30	6	5.2	25.3
4	108,756	2013–2016	Luzerne	448	333	1.4	18.1
5	184,572	2010–2012	Dauphin, Cumberland and York	454	338	1.3	17.9

The demographic and health characteristics for the identified five lung cancer clusters are provided in Table 2. Significant differences were observed in median age, percent male, percent African American, per capita income, percent poverty, percent high school graduate or higher, population density, poor mental health, and poor physical health (all *p* < 0.001) between the clustered and non-clustered census tracts. In our analysis, census tracts that were part of the high incidence clusters tended to have residents of lower median age, had a higher percentage of African Americans, residents below the poverty line, be densely populated, and had poorer mental and physical health compared to residents, not in the clusters.

**Table 2:** Summary statistics of demographic and health characteristics for the whole of PA, clusters, and non-clusters

Variable	Whole PA	In a cluster	Not in a cluster
	Median (IQR)	Median (IQR)	Median (IQR)
Median Age	41.9 (9)	38.1 (10.9)	42.8 (7.8)
Percent Male	48.9 (4.1)	48 (4.9)	49.1 (3.8)
Percent African American	2.8 (10.2)	11 (47.6)	1.9 (6.0)
Percent Asian	0.9 (3.3)	1.3 (4.8)	0.9 (3.0)
Percent Hispanic	2.4 (4.6)	2.4 (2.5)	2.4 (4.7)
Per Capita Income (per 1000 USD)	26.6 (12.3)	24.4 (14.8)	27.4 (11.8)
Median Household Income (per 1000 USD)	51.7 (26.8)	41.9 (26.4)	54.3 (26.6)
Percent Poverty	10.9 (13.0)	18.3 (21.7)	9.5 (9.9)
Percent High School Graduate or less	50.3 (22.9)	50.4 (23.1)	50.3 (22.9)
Percent High School Graduate or higher	90.4 (8.1)	89.7 (10.6)	90.6 (7.5)
Percent Bachelor’s Degree or higher	22.5 (22.1)	21.1 (24.3)	23.1 (21.4)
Percent Graduate Degree	7.7 (9.8)	6.9 (10.7)	7.9 (9.3)
Total Population	3790 (2358)	3269 (2158)	3978 (2368)
Population Density (per sq. mile)	2303.3 (5435.2)	6496.1 (13,005.7)	1430.9 (3571.4)
Percent of poor mental health	14.8 (3.8)	15.8 (5.3)	14.5 (3.5)
Percent of poor physical health	13.0 (4.0)	13.6 (5.6)	12.9 (3.7)

The demographic and health characteristics for each cluster are presented in Table 3. The cluster located in the southeast area of Pennsylvania, Cluster 1, had the highest percent African American (median = 45.4%, IQR = 73.2) and population density (median = 17,785.9, IQR = 15,192.9). Cluster 3 in Mifflin County showed the lowest per capita income (median = 16.5%), the highest percent poverty (median = 29.3%), and poor mental and physical health (median = 19.3% and 17%, respectively).

**Table 3:** Summary statistics of demographic and health characteristics for the five clusters

	Cluster 1	Cluster 2	Cluster 3	Cluster 4	Cluster 5
No. of census tracts	326	410	1	38	47
Variable	Median (IQR)	Median (IQR)	Median (IQR)	Median (IQR)	Median (IQR)
Median Age	33.4 (7.28)	41.5 (9.3)	33.7 ( -)	41.6 (9.1)	39.1 (6.2)
Percent Male	47.5 (6.3)	48.3 (4.5)	51.4 ( -)	47.9 (2.3)	48.1 (4.8)
Percent African American	45.4 (73.2)	4.7 (16.3)	1.5 ( -)	4.4 (8.9)	14.6 (35.2)
Percent Asian	3.3 (8)	0.5 (2.1)	0.5 ( -)	1.2 (1.9)	1.4 (4.6)
Percent Hispanic	4.8 (6.4)	1.3 (2)	3.2 ( -)	4.5 (8.9)	7.6 (8.4)
Per Capita Income (per 1000 USD)	18.8 (14.5)	26.3 (12.4)	16.5 ( -)	21.7 (6.8)	26.4 (11.0)
Median Household Income (per 1000 USD)	35.0 (27.9)	45.8 (24.7)	32.1 ( -)	38.7 (17.0)	50.6 (21.8)
Percent Poverty	28 (24.8)	14.2 (16.6)	29.3 ( -)	20.6 (14.2)	11.7 (16.3)
Percent High School Graduate or less	54.8 (28.5)	47.3 (21.5)	64.4 ( -)	55 (10.6)	47 (18.9)
Percent High School Graduate or higher	84 (6.1)	92.0 (7.2)	86.7 ( -)	89 (6.5)	89.9 (7.4)
Percent Bachelor’s Degree or higher	17.7 (31.3)	24.4 (22.7)	9.5 ( -)	17.8 (7.0)	22.3 (17.3)
Percent Graduate Degree	5.8 (14.5)	8 (9.9)	3.6 ( -)	6 (4.1)	6.9 (7.6)
Total Population	3745 (2067)	2814 (1998)	3616 ( -)	2500 (1504)	3833 (1836)
Population Density (per sq. mile)	17,785.9 (15,192.9)	3690.4 (5030.7)	4417.3 ( -)	4867.6 (4365.2)	3141.0 (4998.5)
Percent of poor mental health	18.1 (6.7)	14.8 (3.8)	19.3	16.9 (3.1)	15.4 (2.9)
Percent of poor physical health	14.2 (7.5)	13.2 (4.3)	15.4	2.8 (2.8)	12.7 (3.2)

Note that Cluster 3 only has 1 census tract so no IQR can be calculated that is the measurement of difference between the third and the first quartiles

## Discussion

In this study, we aimed to identify spatio-temporal clusters of high lung cancer incidence in Pennsylvania over an 8-year period (2010–2017). Overall, the age-adjusted incidence rates and the RR of lung cancer decreased from 2010 to 2017 with no statistically significant space and time interaction, as shown by the parallel of RR estimates over time. Using census tract as the unit, the analyses identified five statistically significant clusters. The most likely cluster was in the southeastern region of Pennsylvania from 2010 to 2013: Delaware, Montgomery, and Philadelphia Counties, and was the most populated; the second cluster concurrently was in southwestern Pennsylvania and overlapped the largest number of counties: Allegheny, Fayette, Greene, Washington, and Westmoreland. Two other clusters, one in Mifflin County (2014–2016) and another in Luzerne County (2013–2016) were limited to years toward the middle of the study period. A final cluster from 2010 to 2012 included Dauphin, Cumberland, and York Counties. in the south-central part of Pennsylvania. The RR ranged from 1.22 for Cluster 2 (2010–2013) to 5.21 for Cluster 3 (2014 to 2016). One of the secondary clusters in the Mifflin County revealed the highest risk ratio of lung cancer compared to the other four clusters, associated with the lowest per capita income (median = 16.51%) and the highest percent poverty (29.3% below poverty line), and the poorest physical and mental health (17% and 19.3%, respectively). The primary economic activities in this area in the past, included the manufacturing of steel [23], machinery [24], and textiles [25], which are often accompanied by adverse environmental impacts and may have contributed to the increased risk of lung cancer. Radon, a known risk factor for lung cancer [26], is also shown to have higher levels in the Mifflin County For example, zip codes in the Mifflin County area were discovered to have high radon levels in homes in the past (e.g., the average radon concentrations were 5.2 pCi/L (first floor) and 11.2 pCi/L (basement) in 17,044, 16.7 pCi/L (first floor), and 20.4 pCi/L (basement) in 17,084). These measurements are much higher than the PA average indoor concentrations of 3.6 and 7.1 pCi/L (first floor and basement, respectively) and above the US EPA standard of 4 pCi/L [27]. Our analyses of socioeconomic and health characteristics suggested that the census tracts with the following characteristics were more likely found in a lung cancer cluster: higher percent African American, lower per capita income, higher percent poverty, and higher population density. Differences in these demographic characteristics reflect that disadvantaged communities are more likely to be exposed to environmental pollutants known to be lung cancer risk factors due to, for example, proximity to manufacturing facilities or hazardous waste sites, traffic, or living in houses with high radon values.

Similar spatio-temporal analyses of lung cancer cases performed by others for the U.S. states of Kentucky [28] and Maine [29] also found results like our findings. For example, the reports by Christian and colleagues that analyzed the spatial and temporal distributions of lung cancer histological types in Kentucky using Scan statistics also found that high risk clusters were near the major metropolitan area and/or overlapped with areas of high poverty. Although our analysis did not distinguish histology types, the locations and characteristics of the clusters found in the current and other studies have further demonstrated the importance of environmental and socioeconomics factors for lung cancer.

### Limitations and future work

One factor that may affect the results is the size and shape of the study area, known as zoning effect [30]. This is a relevant effect on the results of spatial analysis that may change depending on the unit of the analysis. The zoning effect arises when the number of the spatial units of measure remains the same but changes in their relative structure (unit boundaries and shape) generate different analytical results. The scaling parameters in SaTScan may also produce different results depending on scan window sizes, so multiple scans can be performed at various circle sizes [31]. Known as the aggregation effect, the choice of unit for analysis (census tract) may result in the loss of statistical power to detect clusters, and different results may be obtained if other units such as census block groups were selected [32]. Another methodological limitation is the adjustment for age for the clustering analysis which was done using an internal standardization method such that age distribution for the study area was used as the reference population rather than an external standard population (e.g., standard million population). Additionally, lung cancer cases were combined from different stages, histological subtypes and were not stratified by sex. Data on other risk factors such as smoking, occupational and residential history were also not available on the census tract level to allow comparisons between clusters and non-clusters in the current analysis. Smoking is the single highest risk factor for lung cancer. If the smoking data were available, we would have hypothesized that the census tracts in the clusters will have a higher smoking prevalence than the census tracts outside the clusters. This hypothesis was supported indirectly by the results in Table 2 showing the census tracts that were part of the clusters tended to have more residents living below the poverty line, lower-income, and less educated, and positive associations are known to exist between these variables and smoking [33–36]. Furthermore, mental and physical health variables were self-reported and thus subject to error.

Individuals living in high lung cancer incidence clusters may be more vulnerable to multiple risk factors, e.g., smoking history, which is indicative of the health disparity in the state of Pennsylvania. Educational interventions in counties with a higher incidence of lung cancer are important for promoting public health and other risk mitigation practices. These geographic areas warrant further investigation to potentially identify additional risk factors or unique patterns of cancer stage and histology at the diagnosis, to further address environmental exposures and lung cancer risk in those specific counties. Geographic surveillance may help to identify disparities in disease burden among different regions or communities with high-risk populations that could be targeted for public health interventions. Incorporating spatio-temporal statistical methods, such as cluster detection, into existing disease surveillance activities can provide information about potential cancer clusters. For instance, Cluster 5, which included the counties of Cumberland, Dauphin, and York, had the largest U.S. Amish population. Health among the Amish is characterized by higher incidences of many genetic disorders [37, 38], so these communities could be more susceptible to lung cancer. With more vigilant surveillance, early detection of lung cancer may improve overall survival.

## Conclusions

Spatio-temporal analysis of lung cancer incidence in Pennsylvania has led us to the identification of areas with higher risk of developing the disease. Although the age-adjusted incidence rates and RR of lung cancer decreased over time, five statistically significant clusters were identified over the study period from 2010 to 2017. Our analysis also demonstrated significant differences in demographic characteristics including percent African American, percent poverty, and population density between those census tracts that were part of the identified clusters versus those that were not. Poorer mental and physical health were also associated with clustered areas. These geographic areas with increased cancer risk factors require further environmental monitoring and screening efforts to reduce exposures and detect lung cancer at an earlier stage.

## Data Availability

The original data from the Pennsylvania Cancer Registry are not available for redistribution. Summary data generated by the authors are available from the corresponding author on reasonable request.

## Abbreviations

ACS: American Community Survey
BRFSS: Behavioural Risk Factor Surveillance System
CAR: Conditional autoregressive
CDC: Centers for Disease Control and Prevention
ICD: International Statistical Classification of Diseases
IQR: Interquartile range
LLR: Log likelihood ratio
NAACCR: North American Association of Central Cancer Registries
PA: Pennsylvania
pCi/L: Picocuries per liter of air
PCR: Pennsylvania Cancer Registry
R-INLA: R-integrated nested Laplace approximation
RR: Relative risk
SIR: Standardized incidence ratio
